# Unusual Presentation of Case of a Lung Carcinoid Tumor With Ectopic Adrenocorticotropic Secretion (EAS) Associated With Acute Weight Gain and Peripheral Edema

**DOI:** 10.7759/cureus.63619

**Published:** 2024-07-01

**Authors:** Mahmoud Abouibrahim, Mizanour Rahman, Mansoor Zafar, Stefano Berliti, Kadir Hacikurt, Umesh Dashora, Periasamy Sathiskumar

**Affiliations:** 1 Internal Medicine, Conquest Hospital, East Sussex Healthcare NHS Trust, Hastings, GBR; 2 Diabetes and Endocrinology, Conquest Hospital, East Sussex healthcare NHS Trust, St Leonards-on-sea, GBR; 3 Gastroenterology/General Internal Medicine, East Surrey Hospital, Surrey and Sussex Healthcare NHS Trust, Redhill, GBR; 4 Acute Medicine, Conquest Hospital, East Sussex Healthcare NHS Trust, St Leonards-on-sea, GBR; 5 Radiology, Conquest Hospital, St Leonards-on-sea, GBR; 6 Diabetes and Endocrinology/General Internal Medicine, Conquest Hospital, East Sussex Healthcare NHS Trust, St Leonards-on-sea, GBR; 7 Diabetes and Endocrinology, Conquest Hospital, East Sussex Healthcare NHS Trust, St Leonards-on-sea, GBR

**Keywords:** carcinoid tumor, low dose dexamethasone suppression test, high dose dexamethasone suppression test, ectopic acth-producing tumor, acth secreting tumor

## Abstract

Ectopic adrenocorticotropic secretion (EAS) is classically related to small-cell lung cancer but is caused by a wide variety of tumors. In approximately one-fifth of cases, the cause remains unidentified. Excess adrenocorticotropic hormone (ACTH) leads to Cushing’s syndrome, and the presentation can be due to biochemical derangements such as hypokalemia and hyperglycemia. Alternatively, it may manifest with secondary symptoms such as weight gain, hypertension, skin thinning, abdominal striae, and/or psychotic manifestations. The diagnosis is established through dynamic testing after confirming excess cortisol and ACTH levels. Imaging is then used to identify the hormonally active lesion. Controlling hypercortisolism with steroidogenesis inhibitors is the initial step before proceeding to definitive treatment. Ideally, tumor resection, if possible, but bilateral adrenalectomies are considered in cases not amenable to curative surgery.

## Introduction

Ectopic adrenocorticotropic secretion (EAS) constitutes a rare condition, responsible for less than 17% of all Cushing’s syndrome (CS) cases [1]. It can result from excess hormonal secretion caused by small benign tumors or widespread metastatic disease. There are instances where the culprit lesion remains undetected even after conducting exhaustive investigations [1,2]. Symptoms are mainly due to hypercortisolemia, which can cause a wide range of presentations including hypokalemia, hyperglycemia, hypertension, weight gain, abdominal striae, skin bruises, hirsutism, menstrual irregularities, decreased libido, facial plethora, and psychiatric manifestations. The diagnosis poses a challenge as it requires hormonal dynamic testing and radiological scans to accurately diagnose EAS and identify the ectopic source, which remains occult in approximately 20% of cases [3]. EAS carries a high risk of mortality and morbidity if left untreated. Therefore, localizing the source of adrenocorticotropic hormone (ACTH) and removing the lesion contribute to disease remission in 80% of cases, with a better overall prognosis [4]. In our case, we discuss an instance of EAS with an unusual presentation of acute severe peripheral edema and significant weight gain.

## Case presentation

A 76-year-old man was referred by the general practitioner (GP) to the Same Day Emergency Care Unit (SDEC) with worsening bilateral leg swelling of unknown cause, fatigue, dizziness, and a significant weight gain of about 10 kilograms (kg) over 10 days. His blood tests subsequently showed evidence of hypokalemia, which was exacerbated by diuretics prescribed by his GP to treat fluid overload. His past medical history included a left nephrectomy performed around 20 years ago for renal cell carcinoma, chronic obstructive pulmonary disease, hypothyroidism, and a history of active smoking (10 cigarettes per day for 40 years).

Upon examination, he was found to have peripheral fluid overload in both upper and lower limbs, causing pitting edema. His lungs were clear on auscultation, heart sounds were normal, and neck veins were undetectable. The abdominal examination revealed abnormal purplish scar tissue on a previous nephrectomy scar and facial plethora. His systolic blood pressure readings ranged between 160 millimeters of mercury (mmHg) and 195 mmHg. A chest x-ray showed clear lung fields with a normal cardiac shadow. An electrocardiogram (ECG) showed sinus rhythm with an incomplete right bundle branch block. Pro-brain natriuretic peptide (pro-BNP) came back raised at 1,301 nanograms per liter (ng/L), and a cardiology opinion was sought regarding the possibility of a heart failure diagnosis. However, after a specialist review and an Echocardiography study, a heart failure diagnosis was ruled out. Other differential diagnoses included hepatic or renal causes, which were excluded due to normal renal and liver functions with no evidence of organ failure.

**Figure 1:**
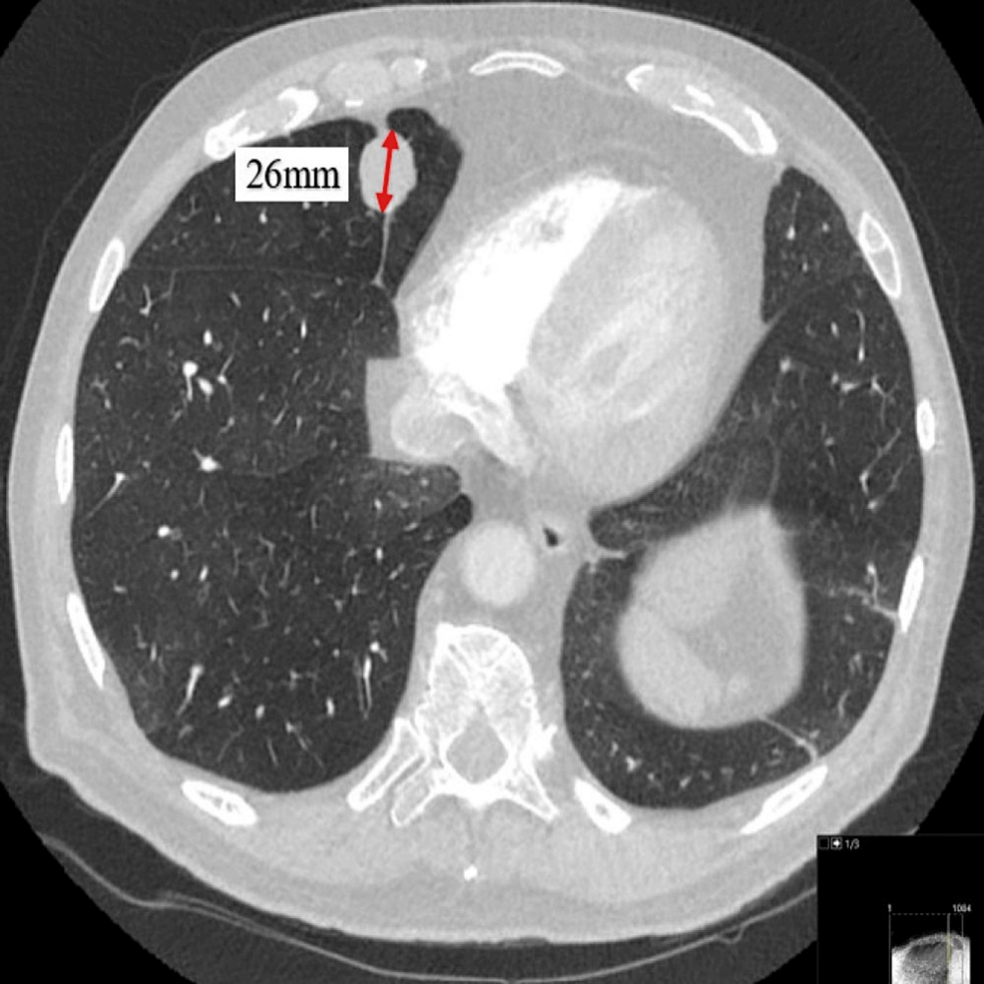
Axial computed tomogram (CT) image on lung window demonstrating an irregular well-circumscribed nodule in the medial segment of the right middle lobe measuring 26 mm (red arrow).

At this stage, a random cortisol test was requested and came back raised at 1,607 nanomoles per liter (nmol/L). Based on the clinical picture, biochemical results, and after reviewing his medications list, an endogenous source of hypercortisolemia was suspected to be the cause of the presentation. Due to his previous cancer history, a computed tomogram (CT) scan of the chest, abdomen, and pelvis with contrast was arranged, which was queried for a 26-millimeter (mm) lesion in the right middle lobe of the lung (Figure 1).

After discussing with the endocrine team, further investigations were requested, including a 9 AM cortisol test, adrenocorticotropin hormone (ACTH), urinary free cortisol in 24hrs and a full anterior pituitary profile. Following a raised 9 AM cortisol result, a 1 mg overnight dexamethasone suppression test (ONDsT), followed by low dose dexamethasone suppression test (LDDsT) and high dose dexamethasone suppression test (HDDsT), both tests showed raised unsuppressed cortisol levels (Table 1).

**Table 1:** Blood test with cortisol level profile. *Overnight dexamethasone suppression test (ONDsT) (1mg) **Low dose dexamethasone suppression test (LDDsT) (0.5mg 6hry for 48hrs) ***High dose dexamethasone suppression test (HDDsT) (2mg 6hry for 48hrs) ACTH: adrenocorticotropin hormone

Test	Result	Unit of measurement	Reference range
Baseline 9 am cortisol	1440	Nanomoles/liter (nmol/L)	137-429
Baseline Plasma ACTH	816	Nanograms per liter (ng/L)	Up to 50
Cortisol level after ONDsT*	1283	Nanomoles/liter (nmol/L)	137-429
Cortisol level after LDDsT**	1305	Nanomoles/liter (nmol/L)	137-429
Cortisol level after HDDsT***	1251	Nanomoles/liter (nmol/L)	137-429
Urinary free cortisol in 24 hours	16930	Nanomoles/24hrs (nmol/24hrs)	Up to 200

Other notable abnormal results showed low gonadotrophins with low testosterone and suppressed free thyroxine (Table 2).

**Table 2:** Gonadotropin level profile. * 5-Hydroxy indoleacetic acid. IGF-1: insulin-like growth factor 1, LH: luteinizing hormone, FSH: follicle-stimulating hormone, TSH: thyroid-stimulating hormone, FT4: free-thyroxine 4, FT3: free tri-iodothyronine 3

Test	Result	Reference range
Serum Prolactin	214 Mu/L	86-324
IGF-1	8.0 nmol/L	6.7-24.1
LH	0.8 IU/L	1.7-8.6
FSH	0.8 IU/L	1.5-12.4
Testosterone	3.19 nmol/L	6.68-25.70
TSH	2.25 Miu/L	0.27-4.20
FT4	10 pmol/L	11-22
FT3	1.2 pmol/L	3.1-6.8
24 hrs urinary metanoradrenaline	1.6 µmol/24hr	Up to 3.7
24 hrs urinary metadrenaline	Undetectable	
24 hrs urinary 5HIAA*	45 µmol/24hr	Up to 42

After detecting hypothyroxinemia, the magnetic resonance imaging (MRI) of the pituitary study was arranged to rule out the pituitary cause of the hormonal disturbances. The study showed a normal gland with no suspicious lesions. Further discussion in the endocrine multi-disciplinary team (MDT) meeting suggested the provisional diagnosis of an ectopic source of ACTH, causing ectopic CS. A decision was also made to start the patient on Metyrapone while waiting for further investigations.

**Figure 2:**
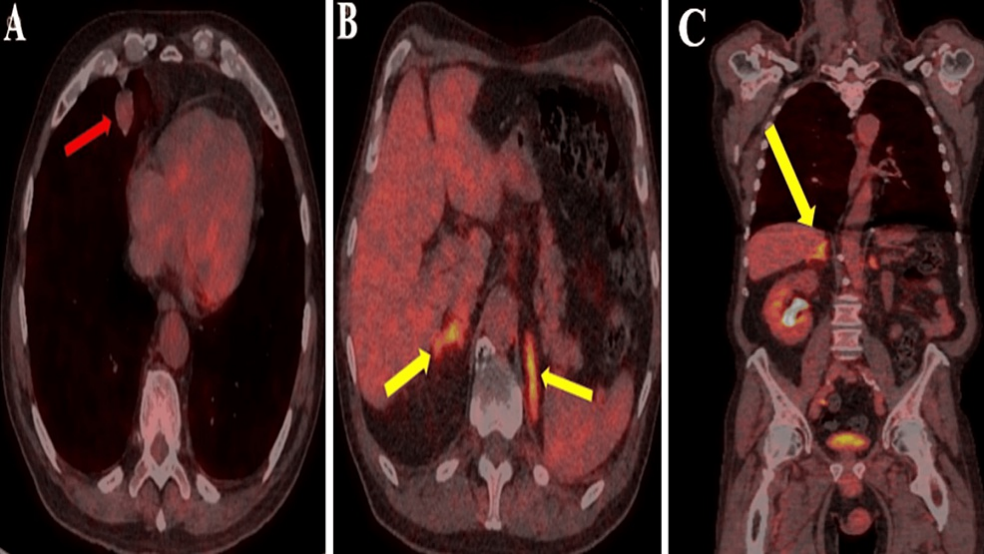
Axial fluorine18-fluorodeoxyglucose positron emission tomogram computed tomogram (FDG PET CT). (A) Right middle lobe pulmonary nodule with low-grade uptake of activity (Herder faint) (red arrow). (B, C) Axial and coronal FDG PET CT images demonstrating high-grade uptake within bilateral prominent adrenal glands with no discrete nodules (yellow arrows).

Additionally, the patient was referred to the respiratory team, who arranged a fluorine18-fluorodeoxyglucose positron emission tomogram CT (FDG PET CT) scan and CT-guided lung biopsy. The FDG-PET scan report indicated low-grade uptake in the lung lesion with slightly bulky adrenal glands with no discrete nodules (KS(SH1)) (Figures 2A-2C).

**Figure 3:**
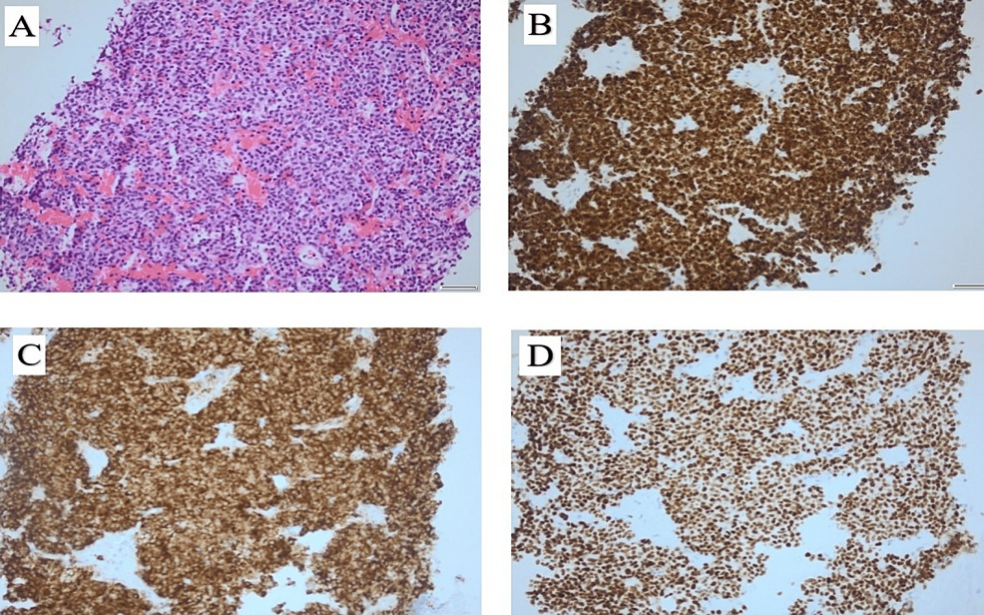
Histology images, lung biopsy. (A) Hematoxylin and eosin (H&E) staining showing uniform population of small round cells with minimal atypia, 40x. (B) Tumor cells positive for Cytokeratin monoclonal antibody AE1/AE3 stain, 40x. (C) Tumor cells positive for synaptophysin (integral membrane protein of small synaptic vesicles in brain and endocrine cells) stain, 40x. (D) Tumor cells showing nuclear positivity for thyroid transcription factor-1 (TTF-1) immunohistochemical stain, 40x.

At this stage, the differential diagnosis included bilateral adrenal metastases or adrenal hyperplasia. The lung biopsy results later confirmed a well-differentiated neuroendocrine carcinoid lesion. A tumor of the neuroendocrine origin (Figures 3A-3D).

On Metyrapone, gradually the patient’s symptoms improved markedly with a resolution of the peripheral edema. The patient’s weight decreased to 87 kg from 102 kg at presentation. There was restoration of normal blood pressure readings and anti-hypertensives were stopped with blood tests showing correction of the hypokalemia and normalization of the cortisol day profile (Table 3).

**Table 3:** Cortisol level post-Metyrapone administration.

Time	Cortisol day study before Metyrapone (nmol/L)	Cortisol day study after Metyrapone (nmol/L)
9:00 AM	1,750	353
2:00 PM	1,681	157
6:00 PM	1,568	214
11:00 PM	1,295	168

**Figure 4:**
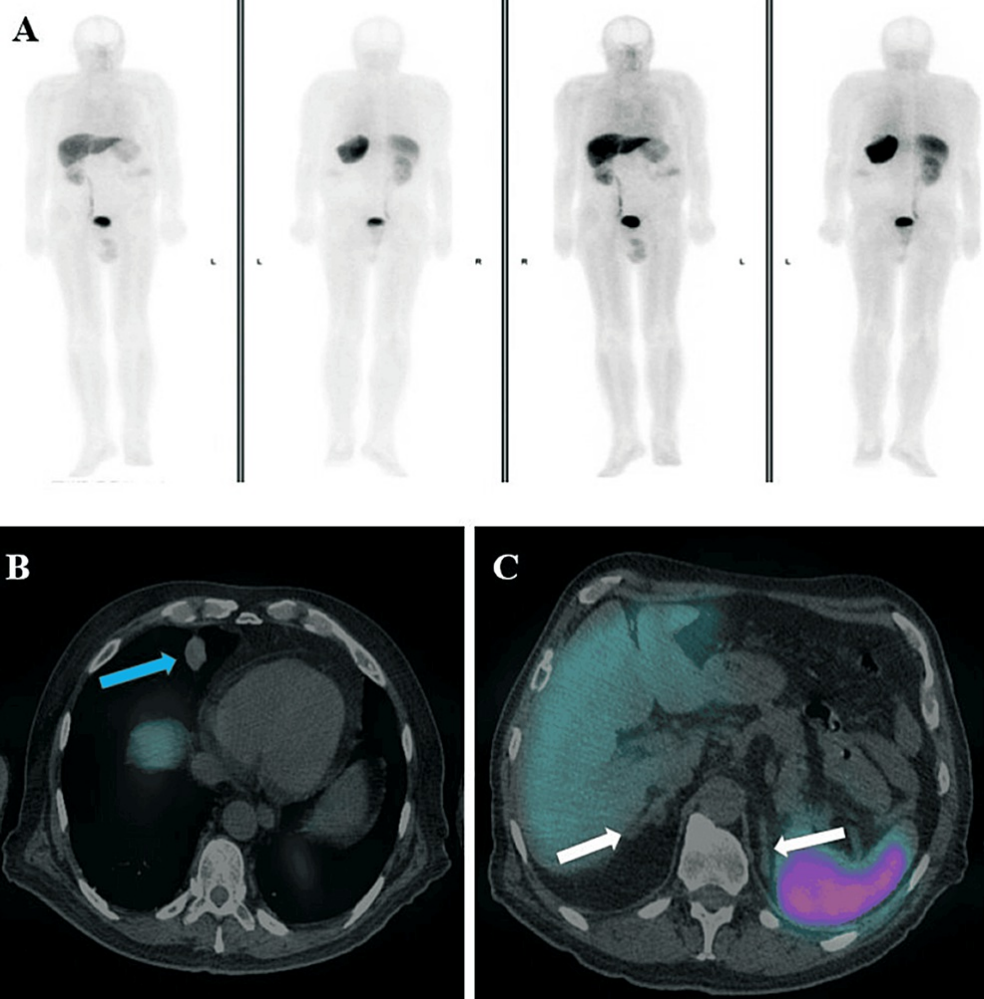
Octreotide single photon emission computed tomography and computed tomogram (SPECT CT) whole body maximum intensity projection (MIP) with no significant uptake (A) and planar trans-axial CT fusion images at 30-minute delay showing no significant tracer uptake in relation to the right middle lobe pulmonary nodule (blue arrow) and adrenal glands (white arrows) (B, C).

Subsequently, an Octreotide (68Ga-DOTA-Nal3/ DOTANOC scan/Somatostatin Receptor Scintigraphy (SRS) scan was arranged and showed only physiological uptake in the adrenal glands with no significant tracer uptake in the right middle lobe pulmonary lesion (Figures 4A-4C).

The case was discussed in a specialized neuroendocrine multi-disciplinary team (MDT) meeting at a tertiary center and the outcome was to proceed to surgery to remove the lung lesion after controlling the hypercortisolemia. The dose of Metyrapone was carefully adjusted according to close monitoring of the cortisol day studies to minimize the risk of adrenal insufficiency. For definitive treatment, he has been referred to thoracic surgeons at a tertiary center for tumor resection and he is currently awaiting his surgery.

## Discussion

EAS leads to excess non-pituitary ACTH, causing CS. Historically, EAS was associated with small-cell lung cancer, as described by Liddle et al. in 1963 [5]. Over the last 60 years, the list of identified causes, including overt and occult tumors causing EAS, has increased significantly [6-8]. EAS is a rare disease with a poor prognosis, occurring in less than 6% of all neuroendocrine neoplasms. These often originate in the lungs, thyroid, stomach, and pancreas. Distant metastasis can be identified in 15% of cases at the time of diagnosis [9-11].

Lung carcinoid tumors can be responsible for secreting many hormones and hormone-like peptides causing a wide range of symptoms and conditions such as 5-hydroxy indoleacetic acid (5-HIAA) causing carcinoid tumors which are twice as common compared with neuroendocrine-induced EAS, other hormones include growth hormone-releasing hormone (GHRH), anti-diuretic hormone (ADH), gastrin, pancreatic polypeptide, human chorionic gonadotropin (HCG) and chromogranin-A [12].

The main presenting features in EAS are secondary to hypercortisolemia, which may lead to weight gain, hyperglycemia, hypertension, muscle weakness, hirsutism, hypokalemia, osteopenia, bruising, infections, edema, hyperpigmentation, and psychiatric disorders [13]. If left untreated hypercortisolemia leads to significant morbidity and mortality mainly secondary to cardiovascular complications such as myocardial infarctions and stroke [14].

Diagnosing EAS requires dynamic non-invasive testing after establishing hypercortisolemia with high serum cortisol levels, 24-hour urinary free cortisol, a 1 mg ONDsT, and/or midnight salivary cortisol, along with high serum ACTH. This is then followed by an LDDsT, further testing with a corticotropin release hormone (CRH) stimulation test, a vasopressin stimulation test, or an HDDsT to confirm or rule out CS. All patients with ACTH-dependent CS should be offered an MRI pituitary and CT scan of the thorax, abdomen, and pelvis to assess for any growth before proceeding to any invasive testing [15-17]. According to one study, in 19% of EAS cases, the ACTH source is not identified after exhaustive investigations. For these cases and for cases with pituitary lesions <6mm, bilateral inferior petrosal sinus sampling (BIPSS) can be an option, with high sensitivity and specificity (94%) [16,18]. Nuclear imaging plays a crucial role in localizing and ruling out distant lesions that could be missed in conventional CT scans. Octreotide scans and FDG-PET scans are widely used in most cases to assess for neuroendocrine tumors and metastatic lesions. In occult cases, a gallium-PET scan has been reported to be more sensitive, and in one study, it revealed all occult cases [17].

Managing EAS initially aims to control the excess cortisol by administering steroidogenesis inhibitors such as metyrapone or ketoconazole with close monitoring of blood and cortisol profile to avoid adrenal insufficiency. Treatment should start in parallel with the ongoing investigation once the EAS diagnosis is confirmed, even if the localization is still incomplete or unsuccessful [18]. The treatment of choice is tumor resection where possible. For cases when targeted surgical removal is not an option, bilateral adrenalectomy with long-term replacement of glucocorticoids and mineralocorticoids is considered [10,18]. In cases with distant metastasis, chemotherapy could be an option, with studies showing poorer outcomes in cases of small-cell lung cancer (SCLC) stage 4 when accompanied by EAS [19].

## Conclusions

Acute weight gain, accompanied by significant peripheral edema, could indicate a state of hypercortisolism and should be suspected if other more common causes cannot be identified. Carcinoid tumors with neuroendocrine differentiation could be the culprit in EAS cases. EAS can cause a wide range of clinical and biochemical derangements and carries a high risk of morbidity and mortality if left untreated. Diagnosis poses a challenge as it requires dynamic hormonal testing and different imaging modalities. Steroidogenesis inhibitors, such as Metyrapone, are used to normalize cortisol levels before proceeding to surgery.
